# Excision and Primary Anastomosis for Bulbar Urethral Strictures Improves Functional Outcomes and Quality of Life: A Prospective Analysis from a Single Centre

**DOI:** 10.1155/2019/7826085

**Published:** 2019-01-23

**Authors:** Pieter D'hulst, Michael S. Floyd, Fabio Castiglione, Kathy Vander Eeckt, Steven Joniau, Frank Van der Aa

**Affiliations:** Department of Reconstructive Urology, University Hospitals Leuven, Herestraat 49, 3000 Leuven, Belgium

## Abstract

**Background:**

Excision and primary anastomotic (EPA) urethroplasty remains the gold standard definitive treatment for short urethral stricture disease. For patients, postoperative erectile function and quality of life are the main goals of the surgery. Patient-reported outcome measures (PROMs) are therefore of major importance.

**Objective:**

The objective of this study was to prospectively analyse functional outcomes and patient satisfaction.

**Design, Settings, and Participants:**

We prospectively evaluated 47 patients before and after EPA from August 2009 until February 2017. The first follow-up visit occurred after a median of 2.2 months (n = 47/47), with the second and third follow-ups occurring at a median of 8.5 months (n = 38/47) and 20.2 months (n = 31/47). Before surgery and at each follow-up visit, the patients received five questionnaires: the International Prostate Symptom Score (IPSS), the International Prostate Symptom Score with the Quality of Life (IPSS-QOL) score, the Urogenital Distress Inventory Short Form (UDI-6) score, the International Index of Erectile Function-5 (IIEF-5) score, and the ICIQ-Lower Urinary Tract Symptoms Quality of Life (ICIQ-LUTS-QOL) score.

**Surgical Procedure:**

Surgery was performed in all cases using the same standardized EPA technique.

**Outcome Measurements and Statistical Analysis:**

Voiding symptoms, erectile dysfunction, and quality of life were analysed using paired sample t-tests, with a multiple-testing Bonferroni correction. Any requirement for instrumentation after surgery was considered treatment failure.

**Results and Limitations:**

Patients with mild or no baseline erectile dysfunction showed significant decline in erectile function at first follow-up (mean IIEF-5 of 23.27 [standard deviation; SD: 2.60] vs. 13.91 [SD: 7.50]; p=0.002), but this had recovered completely at the third follow-up (IIEF-5: 23.25 [SD: 1.91]; p=0.659). Clinically significant improvements were noted in IPSS, IPSS-QOL-score, UDI-6-score, and ICIQ-LUTS-QOL-score at the first follow-up (p<0.0001). These improvements remained significant at the second and third follow-ups (p<0.0001) for all PROMs. Three of the patients experienced stricture recurrence. The main limitations of this study were incomplete questionnaires, loss to follow-up, and low number of patients.

**Conclusions:**

EPA results in an initial decline in erectile function, but full recovery occurred at a median of 20 months. Voiding improved significantly, and a major improvement in quality of life was noted, which persisted for up to 20 months after surgery.

**Patient Summary:**

This study showed the importance of patient-reported outcome measures in indicating the actual outcome of urethral stricture disease surgery.

## 1. Introduction

Urethral stricture disease has an incidence of 0.6%–0.9% in developed countries [1, 2], and it impacts patients' quality of life significantly [2]. Furthermore, when treated endoscopically, the disease has a high recurrence rate, necessitating repeat procedures with costly repercussions for healthcare [1]. Treatment depends on the aetiology, location, and length of the stricture. To identify all aspects of the stricture anatomy, preoperative assessment is essential, including retrograde urethrography, uroflowmetry, and cystoscopy [3].

The most common initial procedures used to treat short (< 1.5 cm), isolated bulbar urethral strictures are internal urethrotomy and dilatation [4, 5]. However, the recurrence-free rates of these procedures are only 39%–73%; repeated urethrotomies or dilatations have even lower success rates and so are not cost effective [6–9]. For this reason, urethroplasty should be considered the procedure of choice in patients with strictures which have recurred after initial endoscopic management or which fail to meet the criteria for single internal urethrotomy or dilatation [9, 10].

The management of bulbar urethral strictures depends on the length of the stricture and the amount of associated spongiofibrosis. For strictures less than 2 cm in length, excision and primary anastomosis (EPA) has shown excellent long-term results [10]. Longer strictures may require substitution urethroplasty [11], which aims to minimize stricture recurrence and the need for further instrumentation. Although the definition of long-term success and the follow-up methods have varied, EPA has shown an overall high level of success (> 90%) across different series [12, 13].

The impact of urethral strictures and subsequent urethroplasty on sexual function, as well as on voiding, should be evaluated postoperatively. Several larger studies have stated that EPA has no significant long-term impact on erectile function [14, 15]. When erectile dysfunction was reported, it tended to be transient, with full recovery 6 months after surgery [15]. However, some recent prospective series have reported that erectile function is poorer after EPA than after stricturotomy and augmentation [16–18], and many surgeons have therefore ceased using classic transecting EPA in favour of nontransecting EPA or augmented urethroplasty. These findings highlight the need for further prospective studies with validated outcome measures. In 2011, the validated Urethral Stricture Surgery Patient Reported Outcome Measure (USS PROM) was developed to assess patient-centred functional outcomes after urethroplasty [19]. This questionnaire assesses lower urinary tract symptoms (LUTS), general health status, and treatment satisfaction. After 2 years' follow-up, it seemed that the USS PROM could generate adequate patient-centred evidence and establish an international consensus on outcome reporting after urethral reconstruction surgery [20].

The present study aimed to use patient-reported outcome measures (PROMs) to prospectively analyse voiding symptoms, erectile function, and quality of life after classic transecting EPA urethroplasty.

## 2. Materials and Methods

**Figure 1:**
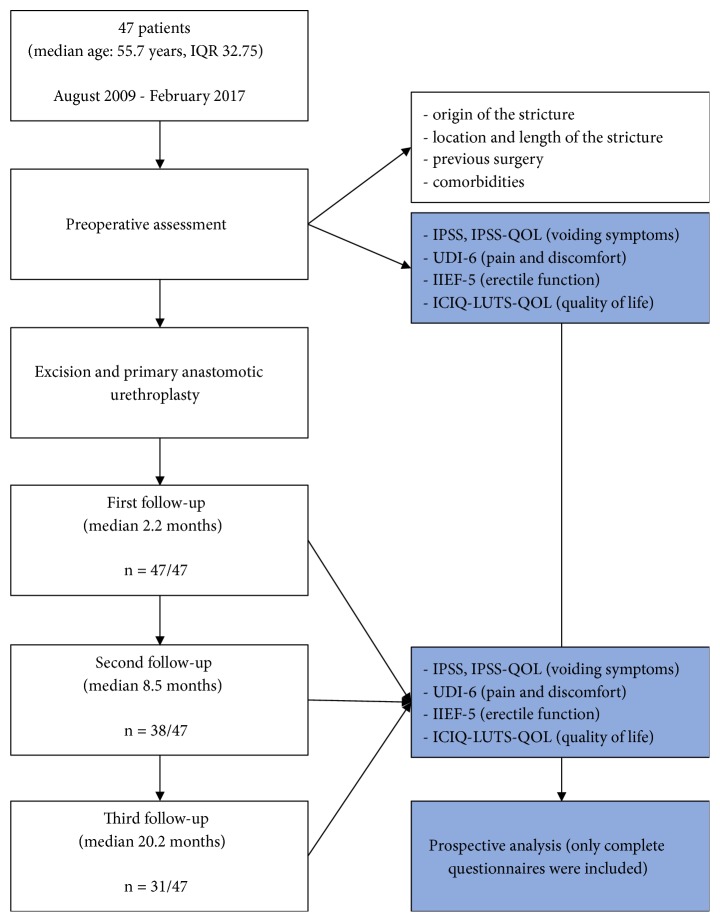
**Flowchart and study design**. *IQR: interquartile range, IPSS: International Prostate Symptom Score, IPSS-QOL: International Prostate Symptom Score with Quality of Life, UDI-6: Urogenital Distress Inventory Short Form score, IIEF-5: International Index of Erectile Function-5 score, and ICIQ-LUTS-QOL: ICIQ-Lower Urinary Tract Symptoms Quality of Life score*.

We prospectively evaluated 47 patients who underwent EPA between August 2009 and February 2017 in University Hospitals Leuven [Figure 1]. All patients provided written and oral informed consent prior to participating in this trial. Men with short (< 2 cm) bulbar urethral strictures were included in the study. Before surgery, the aetiology and characteristics of the strictures were assessed using urethrography, uroflowmetry, urethroscopy (to evaluate the stricture and distal urethra), and urine culture. All procedures were performed by 2 surgeons using the same, standardized classic transecting EPA.

Follow-up visits were organized by the urologist, residents, and secretaries, with the first at 3 months, the second at 9 months, and the third at 18 months. Functional outcomes and impact on quality of life were ascertained by physical examination, uroflowmetry, and validated PROMs. Before surgery and at each follow-up visit, all patients filled out five PROM questionnaires [Figure 1]. Any need for urethral instrumentation following urethroplasty was considered a treatment failure. At each follow-up, complications were recorded using the Clavien–Dindo grading classification system.

The following PROMs were used:

1. The International Prostate Symptom Score (IPSS) [21]
2. The International Prostate Symptom Score with Quality of Life score (IPSS-QOL) [21]
3. The Urogenital Distress Inventory Short Form score (UDI-6) [22]
4. The International Index of Erectile Function-5 score (IIEF-5) [23, 24], with only sexually active men who had intercourse being asked to fill in this questionnaire
5. The ICIQ-Lower Urinary Tract Symptoms Quality of Life score (ICIQ-LUTS-QOL) [25]

The completed questionnaires were scanned into the patients' files and a prospective database was created. All new data was added to this database at each follow-up visit. In February 2017, a total of 47 patients were included. Statistical analysis was performed using the paired-sample t-test, with a multiple-testing Bonferroni correction. To this end, commercially available software (IBM® SPSS® Statistics) was used. The alpha significance level was set at *α* = 0.05 (5%). Kaplan-Meier survival analysis was performed to assess stricture recurrence events in time. The study was approved by the hospital's ethics committee (S55868/B322201319205) and registered at www.clinicaltrials.gov (NCT01982136).

## 3. Surgical Technique

Most procedures were performed under general anaesthesia, with perioperative administration of intravenous cefazolin (2 g). Patients were placed in a modified dorsal lithotomy position. Through a midline perineal incision, sharp dissection was performed to the level of the bulbospongiosus muscle. This muscle was cleaved, and the urethra was dissected circumferentially, distally, and proximally, with sufficient mobility to ensure a tension-free anastomosis. A flexible urethrocystoscopy was performed to assess the stricture location, which was marked by a suture (Monocryl 2/0). At this site, the urethra was transected and the stricture was excised. The urethra was spatulated on both sides within the well-vascularized, healthy tissue. The diseased part was sent for pathological examination. When the stricture was too short to allow traction free anastomosis, the plane between the corpora cavernosa was cleaved to obtain space. An end-to-end anastomosis was performed using eight separate sutures (Monocryl 3/0), and a transurethral 16 Fr. silicone catheter was placed. Haemostasis was induced, the wound was closed in layers, and a compressive bandage was applied. After 24 hours, the compressive bandage was removed, the wound was inspected, and the patient was discharged. When voiding urethrocystography with the transurethral catheter* in situ* showed no leakage after 2 weeks, the catheter was removed.

## 4. Results

A total of 47 patients were included. The first follow-up took place after a median of 2.2 months (n = 47/47), with the second and third follow-ups occurring at a mean of 8.5 months (n = 38/47) and 20.2 months (n = 31/47), respectively [Figure 1]. The patients' median age at surgery was 55.7 years (interquartile range [IQR]: 32.75 years). The median stricture length was 1.0 cm (IQR: 0.7 cm) [Table 1].

The causes of the stricture were trauma (n = 8), infection (n = 1), and iatrogenic (n = 22). In 16 patients, the cause was unknown [Table 1]. The iatrogenic causes were previous transurethral resection (n = 11), catheterization (n = 5), radical prostatectomy (n = 4), and radiotherapy/brachytherapy (n = 2).

All strictures were located in the bulbar urethra (n = 47). A total of 11 patients had undergone no previous surgery, whereas 6 patients had previously undergone only one urethrotomy or dilatation. In total, 29 patients had undergone multiple dilatations or urethrotomies [Table 1], among whom 22 patients had a history of more than 3 previous interventions.

**Figure 2:**
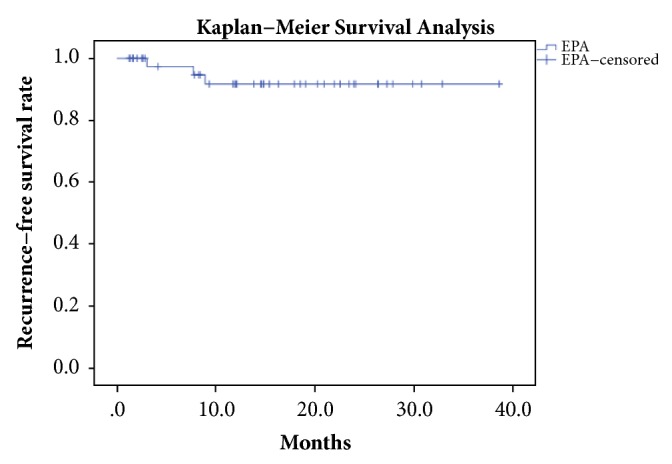
**Kaplan-Meier statistical analysis curve.** *EPA: excision and primary anastomosis*.

In total, 3 stricture recurrences were noted; all occurred within the first 9 months [Figure 2]. Postoperative complications were recorded in 3 patients and consisted of accidental suture-fixing of the catheter (n = 2) and acute bacterial prostatitis (n = 1). Accidental stitching of the catheter should be recorded as a technical failure of surgery. All these complications were observed within the first days and weeks after surgery. The median catheterization duration was 14 days (IQR: 5 days).

## 5. Specific Outcome Measures

### 5.1. Voiding Symptoms (IPSS and Maximal Flow Rate *Q*_*max*_)

**Figure 3:**
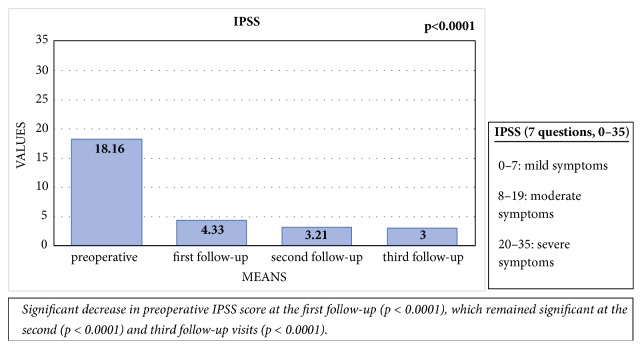
Comparison of the mean preoperative IPSS with the IPSS at the first, second, and third follow-up visits.

The mean preoperative IPSS-score was 18.16 (standard deviation [SD]: 6.35). This had decreased significantly at the first follow-up visit, with a mean score of 4.33 (SD: 3.87) (p < 0.0001). This difference remained significant at the second (p < 0.0001) and third visits (p < 0.0001), with mean scores of 3.21 (SD: 4.46) and 3 (SD: 4.53), respectively. There were no significant differences among the first, second, and third visits in this regard [Figure 3].

There was a significant difference between the preoperative mean Q_max_ (8.43 mL/s, SD: 7.05 mL/s, mean voided volume: 231 mL) and the mean Q_max_ at the first follow-up (25.09 mL/s, SD: 16.61 mL/s, mean voided volume: 272 mL; p < 0.0001). This difference remained significant at the second (p < 0.0001) and third follow-up visits (p < 0.0001), with mean scores of 20.63 mL/s (SD: 11.69 mL/s, mean voided volume: 240 mL) and 23.47 mL/s (SD 9.37, mean voided volume 259 mL), respectively.

### 5.2. Urogenital Distress and Discomfort (UDI-6)

**Figure 4:**
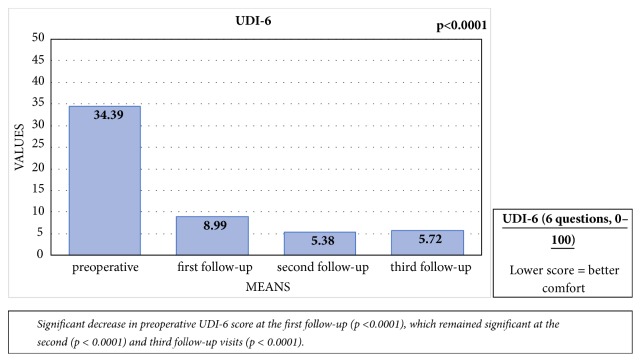
Comparison of the mean preoperative UDI-6 score with the UDI-6 scores at the first, second, and third follow-up visits.

Significant differences were noted between the preoperative mean UDI-6-score of 34.39 (SD: 20.45) and the mean score at first follow-up of 8.99 (SD: 13.66; p < 0.0001). These differences remained significant at the second (p < 0.0001) and third follow-up visits (p = 0.0001), with mean scores of 5.38 (SD: 15.04) and 5.72 (SD: 11.59), respectively. There were no significant differences between the UDI-6 scores at first, second, and third follow-up visits [Figure 4].

### 5.3. Erectile Dysfunction (IIEF-5)

Only 23 of the 47 patients were sexually active before surgery and completed the IIEF-5 questionnaire.

**Figure 5:**
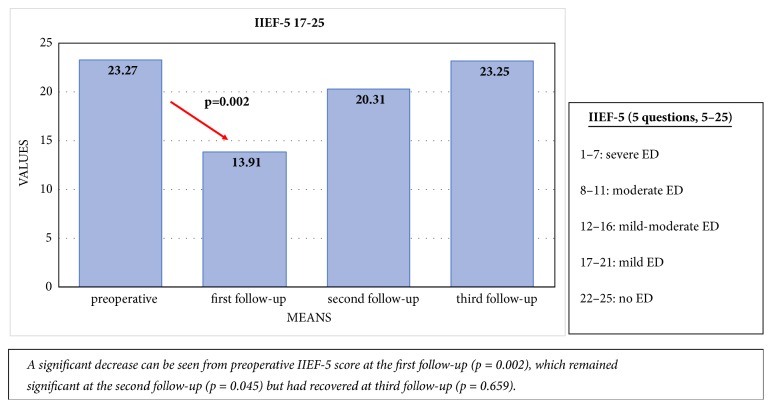
Comparison of the mean preoperative IIEF-5 score with the IIEF-5 scores at the first, second, and third follow-up visits in patients with mild-to-no erectile dysfunction at baseline.

Patients with mild or no baseline erectile dysfunction (IIEF-5: 17–25) had a significant decline in erectile function at the first follow-up (IIEF-5: 23.27, SD: 2.60 vs. 13.91, SD 7.50; p = 0.002; n = 15/23). At the second follow-up, erectile function still differed significantly from preoperative values [IIEF-5: 20.31, SD: 5.15; p = 0.045; n = 15/23). By the third follow-up, a full recovery was seen, and erectile function did not differ significantly from the preoperative value [IIEF-5: 23.25, SD: 1.91; p = 0.659; n = 15/23) [Figure 5].

**Figure 6:**
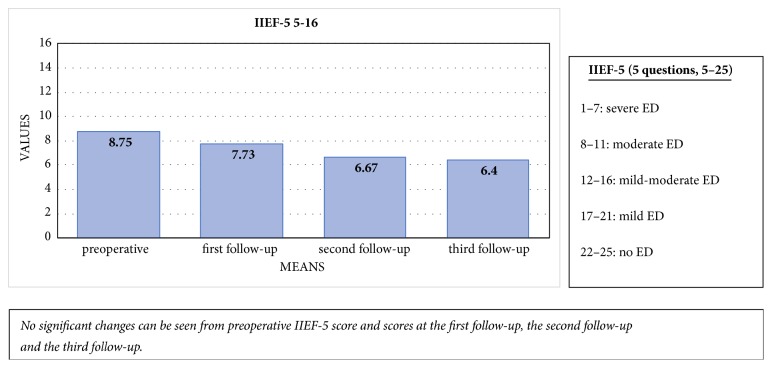
Comparison of the mean preoperative IIEF-5 score with the IIEF-5 scores at the first, second, and third follow-up visits in patients with moderate-to-severe erectile dysfunction at baseline.

Patients with mild/moderate to severe ED (IIEF-5: 5–16) at baseline (n = 8/23) experienced no significant difference in erectile function at the first follow-up [IIEF-5: 8.75, SD: 4.53 vs. 7.73, SD: 2.55; p = 0.453; n = 8/23), second follow-up [IIEF-5: 6.67, SD: 0.82; p = 0.187; n = 8/23), or third follow-up [IIEF-5: 6.40, SD: 1.52; p = 0.477; n = 8/23) [Figure 6].

### 5.4. Quality of Life (IPSS-QOL and ICIQ-LUTS-QOL)

**Figure 7:**
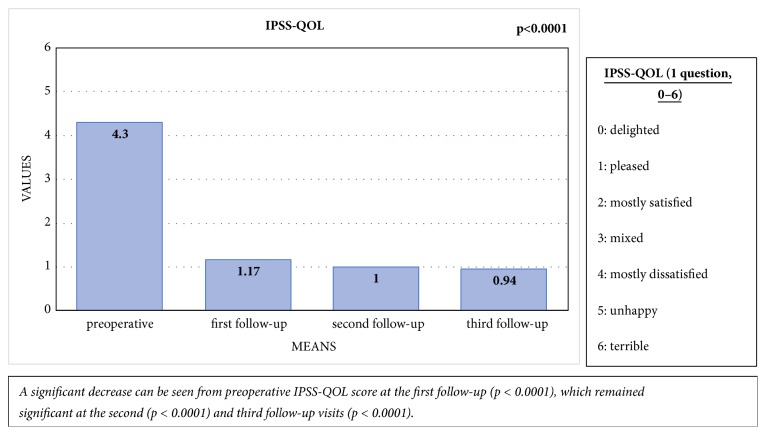
Comparison of the mean preoperative IPSS-QOL score with IPSS-QOL scores at the first, second, and third follow-up visits.

A significant improvement was noted between the preoperative mean IPSS-QOL score of 4.30 (SD: 1.17) and the mean score of 1.17 (SD: 1.03) at the first follow-up visit (p < 0.0001). This improvement remained significant at the second (p < 0.0001) and third follow-up visits (p < 0.0001), with mean scores of 1 (SD: 1) and 0.94 (SD: 1.21), respectively. There were no significant differences between the first, second, and third follow-up visits in this regard [Figure 7].

**Figure 8:**
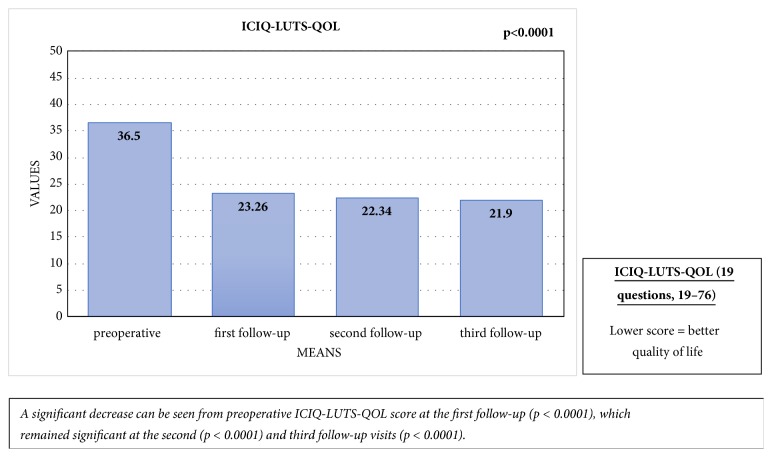
Comparison of the mean preoperative ICIQ-LUTS-QOL score with ICIQ-LUTS-QOL scores at the first, second, and third follow-up visits.

The mean preoperative ICIQ-LUTS-QOL score was 36.5 (SD: 10.34), and it had decreased significantly at the first follow-up visit, with a score of 23.26 (SD: 6.13; p < 0.0001). This improvement also remained significant at the second (p < 0.0001) and third follow-up visits (p < 0.0001), with mean scores of 22.34 (SD: 6.72) and 21.90 (SD: 6.97), respectively. There were no significant differences between the first, second, and third follow-up visits [Figure 8].

## 6. Discussion

### 6.1. Voiding Symptoms and Urogenital Distress

In the present study, EPA led to a significant decrease in IPSS score, as measured at the first, second, and third follow-up visits, indicating an improvement in LUTS. Furthermore, we noticed a significant improvement in Q_max_ after surgery. The main voiding complaints of urethral stricture disease are weak stream, dribbling, and incomplete emptying [26]. The IPSS assesses most of these symptoms. In addition, there was a significant decrease in UDI-6 score during follow-up in the present study, highlighting an improvement in dribbling, incontinence, and pain after surgery. These findings are similar to those described by Jackson et al., who used the USS PROM questionnaire [19, 20].

EPA aims to remove the urethral stricture and associated spongiofibrosis, as well as to reconstruct the urethra with an adequate and sufficient diameter. In the present study, there was a significant decline in IPSS and a significant improvement in Q_max_ after surgery. Therefore, we conclude that EPA resolves obstructive voiding symptoms and improves urinary flow.

### 6.2. Erectile Dysfunction

We noticed a significant decline in the IIEF-5 score at the first follow-up in sexually active patients who had good erectile function before surgery (n = 15/23). This first decline could be attributed to pain and catheterization during the first weeks after surgery [15, 16]. Full recovery of erectile function was seen at the third follow-up. In contrast, patients with moderate to severe erectile dysfunction at baseline experienced no significant difference over time.

This transient decline in erectile function was also described by Erickson et al., with a similar return to baseline erectile function during follow-up [15]. Minimally invasive urethroplasty is becoming more popular, and recent studies have suggested that transection of the corpus spongiosum leads to less favourable outcomes with regard to erectile function [17, 18], perhaps because there is a close anatomical relationship between the bulbar urethra and the erectile innervation [18]. The nontransecting technique preserves the well vascularised underlying spongiosum and thus has a lesser impact on sexual function [18]. Consequently, nontransecting EPA has shown potential benefits. Our data, although involving only a small subgroup of patients with good baseline erections (n = 15/23), showed no differences in erectile function at longer follow-up. Thus, patients with normal erectile function should be counselled before surgery regarding the possibility of early erectile dysfunction, and patients with moderate to severe erectile dysfunction before surgery are unlikely to develop improved erectile function.

### 6.3. Quality of Life

Following reconstruction, patients were pleased with their voiding function, and a significant improvement was noted between the preoperative and postoperative IPSS-QOL scores. We noticed a similar change in the ICIQ-LUTS-QOL questionnaire, which extensively questioned quality of life before and after surgery and explores in detail the impact of different treatment modalities on the patients' lives [25]. Our findings are consistent with a prospective series published by Jackson et al. in 2013, wherein most patients (87%) were satisfied or very satisfied after surgery, with significant improvement in their health state index [20]. Therefore, our data show that EPA has a significant positive impact on quality of life.

### 6.4. Stricture Recurrence and Complications

Three patients experienced stricture recurrence in the present study, and all of these failures occurred in the first 9 months. Kaplan-Meier survival analysis was carried out to ensure that surgical success was correctly reported, since 16 patients were lost to longer follow-up [Figure 2]. At long follow-up, we noticed no new stricture recurrences, and no patients have yet required repeat urethroplasty. Ultimately, we achieved a high recurrence-free rate, and our findings were similar to those of previous studies, which have reported high levels of overall success (> 90%) [12, 13].

Only 3 complications were recorded, and these were also comparable with previous studies [10, 13]. According to the Clavien–Dindo grading classification system, we had 1 Grade II complication (bacterial prostatitis requiring pharmacological treatment) and 2 “Grade IIIb” complications (suture-fixed catheter requiring endoscopic intervention under short general anaesthesia) [27]. All these complications were seen in the first 3 weeks after surgery.

## 7. Strengths and Limitations

The key strengths of this study were its prospective nature and the availability of preoperative values. All the patients were asked to complete the questionnaires as stated above and these data were accurately recorded. However, there were some limitations. It was a single centre series with a relative low number of patients and a short follow-up. Consequently, the study lacked statistical power. Furthermore, several incomplete questionnaires were submitted: 3 at the first follow-up, 3 at the second follow-up, and 5 at the third follow-up. Some patients were also lost to follow-up, mainly because some patients were followed by the referring urologist. In 2011, Jackson et al. published a validated, patient-reported outcome measure to analyse patient satisfaction and relief of symptoms after urethroplasty [19]. In the present study, we did not use this validated questionnaire, because our analysis began in 2009. Thus, to prospectively evaluate our patients, we used other validated questionnaires, as detailed above. The UDI-6 score to assess urinary incontinence, pain, and discomfort was initially established for use in clinical and research studies in women [22]. However, in 2015, this questionnaire was also validated in men [28]. In 2017, Verla et al. published a validated Dutch version of the USS PROM questionnaire [29]. Since our centre is located in the Dutch speaking part of Belgium, this validated questionnaire may have been beneficial, since it also includes questions about the patients' general health status and ejaculatory dysfunction, which were not assessed in this trial.

## 8. Conclusion

At presentation, the questionnaires indicated that the patients had bothersome voiding symptoms and impaired quality of life. After classic transecting EPA urethroplasty, their voiding symptoms had improved significantly, without significant impact on erectile function. Furthermore, we noticed an improvement in quality of life which remained significant for up to 20 months after surgery. This prospective study emphasizes the importance of patient-reported outcome measures when assessing the results of reconstructive urethral surgery. Operative success should not merely be defined in terms of the need for stricture-related interventions, as erectile dysfunction and voiding symptoms contribute to quality of life, and thus to overall surgical success.

## Figures and Tables

**Table 1:** Patient and stricture demographics.

Preoperative characteristics of the study population (47 patients)	
Median age	55.7 years (IQR: 32.75 years)

Median stricture length	1 cm (IQR: 0.7 cm)

Median follow-up (months)	1st follow-up: 2.2 months (IQR: 1.1 months)
	2nd follow-up: 8.5 months (IQR: 2.4 months)
	3rd follow-up: 20.2 months (IQR: 9.4 months)

Stricture location	47/47: bulbar (100%)

Stricture aetiology	8/47: traumatic (17%)
	22/47: iatrogenic (47%)
	16/47: idiopathic (34%)
	1/47: infection (2%)

Previous surgery	29/47: repetitive urethrotomy/dilatation (62%)
	1/47: open surgery + dilatation (2%)
	11/47: no previous surgery (23%)
	6/47: one urethrotomy or dilatation (13%)

IQR: interquartile range.

## Data Availability

The data used to support the findings of this study are available from the corresponding author upon request.
